# Maximizing choreography and performance in artistic swimming team free routines: the role of hybrid figures

**DOI:** 10.1038/s41598-023-48622-3

**Published:** 2023-12-02

**Authors:** Leijiao Yue, Jiawen Zhang, Wenlai Cui, Rui Yang, Jun Yin

**Affiliations:** 1 Physical Education and Training Institute, Capital University of Physical Education and Sports, Beijing, China; 2 School of Swimming and Diving, Beijing Sport University, Beijing, China; 3 School of Dance and Martial Arts, Capital University of Physical Education and Sports, Beijing, China

**Keywords:** Human behaviour, Biological physics

## Abstract

Hybrid figures serve as an important component of artistic swimming, however, no prior research has investigated objective indicators for predicting total scores in competition. This study aimed to identify significance of hybrid figure (HF) elements in predicting total scores in artistic swimming competitions and compare these variables between the Beijing team trials and international official competitions. Eight HF variables were measured in the videos from the international artistic swimming competitions, with calibration and measurement of the variables leg height index and leg angle deviation being performed by means of Kinovea. Multiple linear regression was conducted to predict the total scores based on these variables investigated for the international teams, which showed 5 significant predictors including movement frequency, leg height index, leg angle deviation, mean pattern duration, and rotation frequency. Wilcoxon signed-rank test was used to determine the differences in the variables between the Beijing team and the international teams (top 5), which showed significant difference on movement frequency, leg angle deviation, leg height index and total score. When designing hybrid figures choreography of team free routine in artistic swimming, coaches should prioritize appropriate movement frequency, pattern changes and rotations over excessively long durations. Overly complex HF choreography may lead to a decline on performance in artistic swimming competition.

## Introduction

Artistic swimming (synchronized swimming) is an aesthetic sport combining elements of swimming, dance and gymnastics, all performed to music. Among all different forms in artistic swimming competitions, the team free routine, featuring 8 artistic swimmers, is a prominent category. The team free routine was first introduced in the Olympic Games Sydney 2000, replacing the solo routines performed by a single artistic swimmer^1^. Since then, artistic swimming has been popular around the world and incorporated into various international championships. The world’s powerhouses in artistic swimming traditionally include Russia, China, Ukraine, and Japan, all having accumulated numerous Olympic medals.

In a team free routine, the total score comprises three key elements: execution, artistic expression, and difficulty, which accounting for 30%, 40% and 30% of the total score, respectively^2^. Execution and difficulty scores are mainly determined based on the performance and synchronized level of figures (swimmers in an inverted position and the leg moves following choreography above the water) and patterns (geometric formations created by eight swimmers, including but not limited to squares, rectangles, triangles, straight lines or curve lines), whereas artistic impression scores primarily depend on the variety and creativity in choreography^3^. Similar to other aesthetic sports (such as figure skating and artistic gymnastics), artistic swimming scores are determined by subjective evaluations of a panel of judges while objective measurements, such as distance, time or height in track and field events are not applied^4^. Even though the details of the evaluation criteria and scoring scales are provided by the FINA artistic swimming rules^5^, abstract artistic impression scores remain highly subjective^6^. Thus, this may result in inconsistencies in scoring across different judges or competitions. To objectify scoring in aesthetic sports like figure skating, advanced action recognition approaches have been developed for sports video analysis^7^. Several studies have employed machine learning and deep learning algorithms to predict figure skating competition scores^7,8^. However, these techniques and methods have not yet been used in artistic swimming competitions. Thus, it is necessary to introduce news methods to objectively score the artistic swimming.

A team free routine in artistic swimming consists of hybrid figures (HF), which are sets of choreographed leg movements performed above water, each with its own level of difficulty^9^. As one of the most important components of team free routine, complex and varied HF play a key role in the competition, which are composed of various forms including barracuda, albatross, seagull, ballerina, etc. Several elements of HF in a team free routine can significantly impact the difficulty score, execution score and artistic impression score^5^. Previous study has shown that the duration of HF underwater has a large impact on total score, and judges may prefer HF with long dives in competitions^10^. During international competitions, the team free routines typically have a duration of 3–4 min, with artistic swimmers often spending approximately 50–65% of the total time submerged underwater^11^. Last HF duration, movement frequency and pattern duration may be important factors for difficulty score^5^. For execution score, swimmers should try to reach a vertical position as high as possible when performing two legs vertical position, such as barracuda^12^. For artistic impression score, the main influencing factors may include movement connections—actions created when swimmers link together—and rotations performed on the horizontal plane or axis^5^. In conclusion, there may be several HF elements closely related to total score. However, research on relationship between HF elements and scores or artistic swimming score prediction is limited, the way in which HF elements collectively affect total score is unclear. In other aesthetic sports, previous studies have found that subjective scoring in a competitive diving event can be mathematically modeled, simultaneously the elements commonly assumed to affect dive scoring (like height or angle) actually do affect scoring^13,14^. Therefore, it is significant to explore whether HF elements could impact the total score in artistic swimming.

The present study comprised two parts. The first purpose of this paper was to explore the correlation between the HF elements and total scores in the international official competitions. Secondly, we compared the difference between the Beijing Artistic Swimming Team (Beijing team) to teams in international official competitions to explore potential differences in these HF variables between teams at different levels, aiming to provide suggestions for HF choreography.

## Methods

### Participants

The trials in this study included 10 female artistic swimmers (age 21.3 ± 2.2 years; body height 168 ± 3.27 cm; body mass 50.36 ± 2.33 kg) who had been training in Beijing artistic swimming team for 11 to 14 years. Informed consent was signed prior to the beginning of the trials. The study was conducted in accordance with the ethical principles of the Helsinki declaration for human research. All procedures were approved by the ethics committee of Capital University of Physical Education and Sports (Approval number: 2023A055).

### Design and procedures

This study comprised 2 parts. Initially, it analyzed the video footages of the artistic swimming official competitions that were publicly available online, as previous study had proven video to be a reliable tool for analyzing artistic swimming competition^15^. Videos of team free routines of the international artistic swimming official competitions were downloaded for analysis. All official competitions held between 2015 and 2022 were selected for analysis, including the 16th FINA World Championships 2015, 2016 FINA Olympic Games Qualification Tournaments, Olympic Games Rio 2016, 17th FINA World Championships 2017, LEN European Aquatics Championships 2018, Asian Games Jakarta 2018, 18th FINA World Championships 2019, LEN European Aquatics Championships 2021, FINA Olympic Games Qualification Tournament 2021, Olympic Games Tokyo 2020, and 19th FINA World Championships 2022. In summary, videos from 105 teams participating in 11 international official competitions were incorporated into the study.

**Figure 1:**
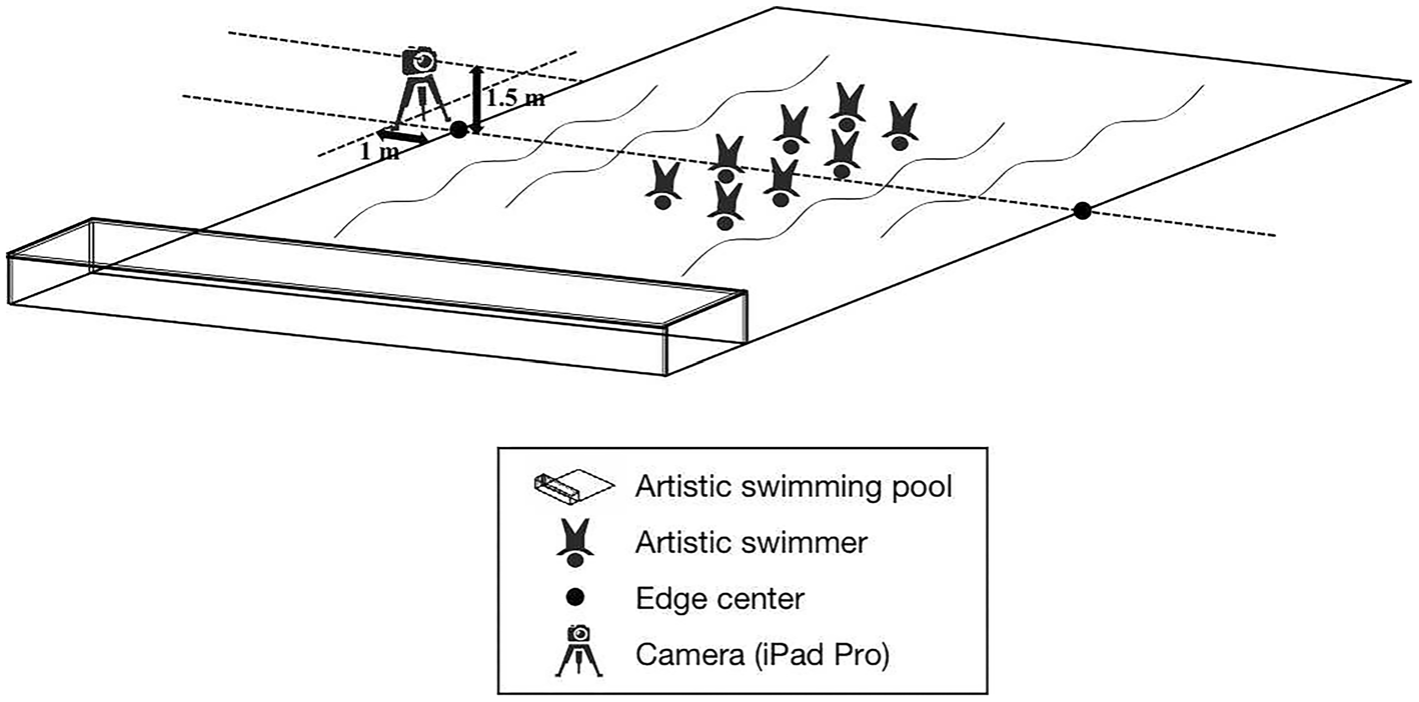
The Camera position for team free routine trials of Beijing Artistic Swimming Team.

Second, this study compared the differences in HF key elements and total score between the Beijing Artistic Swimming Team (Beijing team) and the teams in international official competitions. Beijing team has represented Beijing to won more than ten gold medals in the National Games of the People's Republic of China. Prior to video recording, the participants were fully informed of the study procedures, and all of them signed written informed consent form. Two trials of Beijing team’s team free routines were recorded from the judges’ view^5^ using an iPad Pro (A2228, Apple Inc., Cupertino, CA, USA) at 30 Hz, situated 1 m far away from the swimming pool edge, with the camera axis was set at 1.5 m above the ground (Fig. 1). The trials were conducted during the regular training times, and were scored by 1 International judge (level G) and 8 national judges. Subsequently, the total scores and 8 variables from the Beijing team’s trials were compared with those of the teams in international official competitions (n = 105). Given that Beijing team is one of the highest-level teams in China, the results of the top 5 teams of the international official competitions (n = 30) were used to be compared with Beijing team.

### Measures

The videos were processed using an image of 2D software analysis (Kinovea^®^, Version 0.9.5, USA), which has been used in a previous study on artistic swimming^16^ and showed excellent accuracy in measuring objects at distances and leg degrees for artistic swimming HF. To investigate the relationship between the HF elements and the scores, 8 variables were obtained, including mean HF length, last HF duration, movement frequency, mean pattern duration, rotation frequency, connection frequency, leg angle deviation and leg height index, and the variable descriptions are shown below.

Mean HF duration: this was calculated as the average duration of all sets in one team. Each set of hybrid figures was timed “from breath to breath" − from the last breath before entering the water to the first breath after completing the hybrid.

Last HF duration: the duration of the last set of HF duration was extracted.

Movement frequency: any change in the position or direction of the lower limbs required for the choreography of the routine was considered as a movement^2^. Movement frequency was calculated as the total number of movements and was normalized to the total HF duration for comparison across different trials.

Mean pattern duration: the total number of patterns was counted for each team, and then was normalized to the total HF duration.

Rotation frequency: the total rotation degree on the horizontal plane and horizontal axis was counted and then normalized to the total HF duration.

Connection frequency: the total number of connections was counted for each team, and then was normalized to the total HF duration.

**Figure 2:**
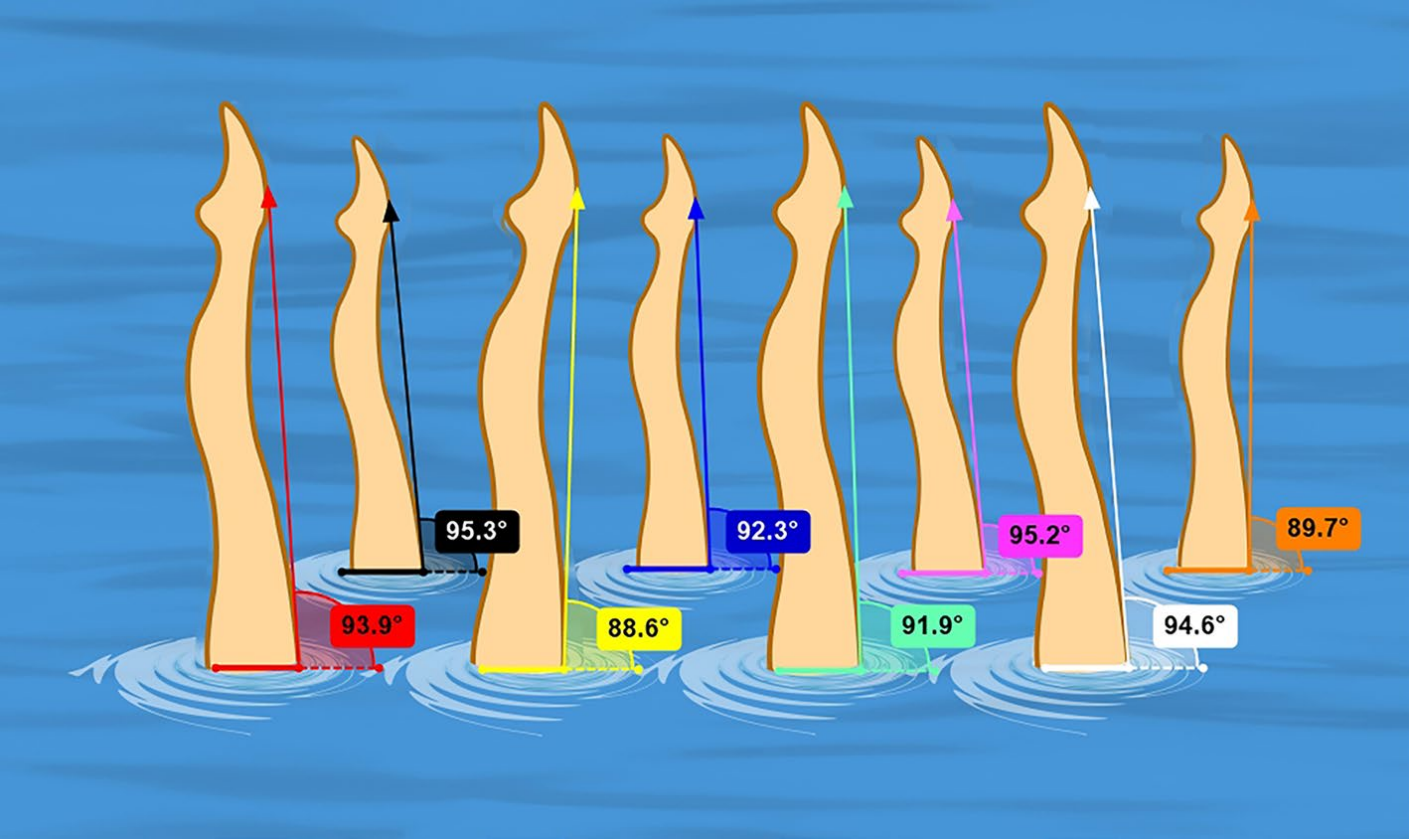
Angle of Vertical Position with one leg (Fishtail or Bent Knee) and measurement of deviation from right angle. Using the Kinovea software, a line was drawn from the most prominent of the instep to the most front point of the thigh above the water. Then the average value of leg angle deviations deviated from right angle of 8 team members was calculated.

Leg angle deviation: to analyze the videos, the first clear image frame that could distinctly show the swimmer’s legs above the water was selected when the leg should be as vertical as possible at a single leg vertical position (Fish tail or Bent Knee). The leg angle was measured between the drawn line and horizontal line. A leg angle of 90° indicates the leg is vertical to the horizontal plane. The average value of leg angle deviations from right angle of 8 team members was then calculated (Fig. 2).

**Figure 3:**
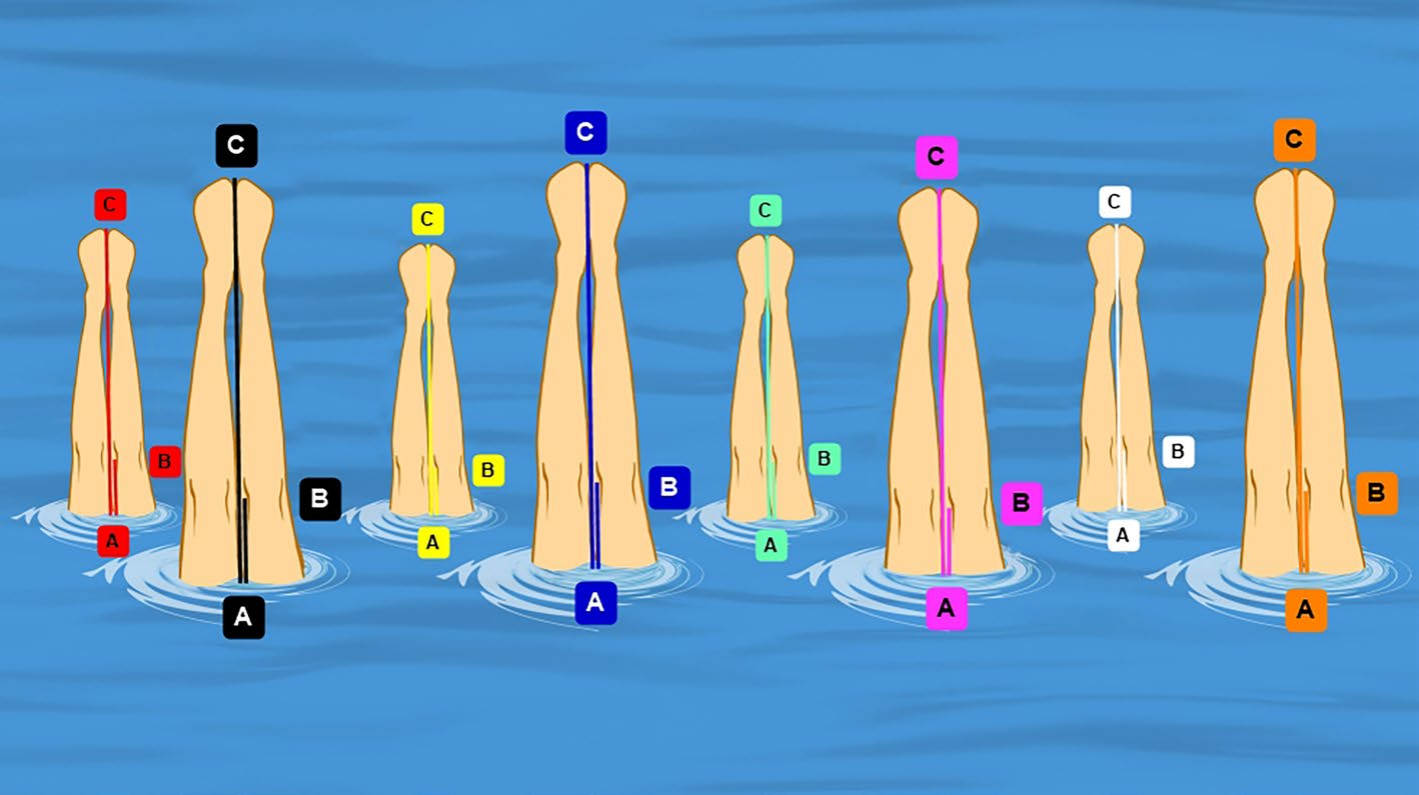
Measurement of the vertical height in the double-legged vertical position, accompanied by the determination of the length ratio between segments AB and AC. For each team member, the length from the water (**A**) to the highest toe (**C**) was measured as AC (in pixels). After that the length from the water to the medial condylis of the femur (**B**) was measured as AB (in pixels).

Leg height index: given that the dimension of the capture volume was not calibrated, the height cannot be directly measured with video analysis, necessitating the proposal of a leg height index. This index was measured for each team member to indicate the leg length above the water at this vertical position. As illustrated in Fig. 3, the length ratio of AB to AC was calculated to get the leg length index. A greater index indicates that the leg reached a higher position in the two legs vertical position. Then the average value of the 8 team members was obtained (Fig. 3).

The same methodologies were employed to analyze the videos of Beijing team’s team free routine. The mean variables of twice team free routine trials were taken for analysis.

### Statistical analyses

Descriptive statistics was calculated and reported as mean ± standard deviation for normally distributed data or as median (interquartile range) for non-normally distributed data. The normality of the data was assessed with Kolmogorov–Smirnov test. In order to assess the reliability of the HF element collection method, a team was randomly selected from each of the 11 international official competitions to serve as the subject for verification. Three Beijing artistic swimmers, who had participated in international competitions, were tasked with collecting HF elements from videos of each of these teams. Inter-rater reliabilities were examined using the intraclass correlation coefficient (ICC) in SPSS (Version 22, IBM Corp, Armonk, USA). The agreement was interpreted as: fair (0.40–0.59); moderate (0.60–0.74), and a good to excellent (0.75–1.00)^17^. An enter multiple linear regression was conducted to predict the total scores based on the eight variables investigated in this research. The sample size estimation was performed using PASS 11.0 software. The significance level (α) was set at 0.05, and the test power (1-β) was established at 0.90. The number of variables in the control set was designated as 3, and the number of variables in the significant test set was determined to be 5. R^2^ was set at 50%, 60%, 70%, and 80%. Upon computation, the minimum sample size required for the multiple liner regression was estimated to range from 14 to 24 cases. Furthermore, the normality assumption for the multiple linear regression was validated by conducting a normality test on the residuals of the regression model. The residuals were found to follow a normal distribution, indicating the fulfillment of the normality assumption. To determine if there were significant differences in the variables between the international teams and those of the Beijing team, different statistical approaches were employed, Wilcoxon signed-rank test was implemented if the data did not satisfy the criteria for normality. Statistical significance was established at the *P *< 0.05 level.

## Results

### Reliability

The reliability of HF element measurements shown in Table 1 was excellent according to the ICC for the mean HF duration (ICC = 0.933, 95% CI 0.830 − 0.980), last HF duration (ICC = 0.982, 95% CI 0.951 − 0.995), mean pattern duration (ICC = 0.36, 95% CI 0.812 − 0.982), movement frequency (ICC = 0.901, 95% CI 0.671 − 0.973), rotation frequency (ICC = 0.959, 95% CI 0.881 − 0.988), connection frequency (ICC = 0.980, 95% CI 0.946 − 0.994), leg height index (ICC = 0.898, 95% CI 0.753 − 0.969), and leg angle deviation (ICC = 0.977, 95% CI 0.939 − 0.993).

**Table 1:** Reliability of assessment method for 8 HF elements.

Variables	Intra-class correlation	95% confidence interval		*p*
		Lower	Upper	
Mean HF duration	0.933	0.830	0.980	< 0.001*
Last HF duration	0.982	0.951	0.995	< 0.001*
Mean pattern duration	0.936	0.812	0.982	< 0.001*
Movement frequency	0.901	0.671	0.973	< 0.001*
Rotation frequency	0.959	0.881	0.988	< 0.001*
Connection frequency	0.980	0.946	0.994	< 0.001*
Leg height index	0.898	0.753	0.969	< 0.001*
Leg angle deviation	0.977	0.939	0.993	< 0.001*

### Value of international teams and top 5 teams in official competitions

Descriptive results of the total scores and the HF variables are shown in Table 2. The mean total score achieved by international teams was 90.0 (9.05), with 8 HF variables were mean HF duration 11.62 (2.43), last HF duration (15.45 ± 3.74), mean pattern duration 5.82 (1.70), movement frequency (1.82 ± 0.17), rotation frequency (41.77 ± 10.01), connection frequency 0.06 (0.12), leg height index (30.77 ± 3.12), and leg angle deviation 4.94 (2.94), respectively. The mean total score achieved by international teams (top 5) was 94.91 ± 2.27, with the corresponding values of 8 HF variables as follows: mean HF duration (12.54 ± 1.83), last HF duration (16.98 ± 4.03), mean pattern duration (5.45 ± 0.93), movement frequency (1.92 ± 0.15), rotation frequency (44.95 ± 10.07), connection frequency 0.10 (0.15), leg height index 32.55 (2.00), and leg angle deviation 4.02 (1.99), respectively.

**Table 2:** Total scores and key variables of international teams in official competitions and top 5 teams in recent 11 international official competitions.

Variables	Value of international teams in official competitions (n = 105)				Value of top 5 international teams in official competitions (n = 30)			
	Mean	SD	Median	IQR	Mean	SD	Median	IQR
Total score	88.36	6.98	90.0	9.05	94.91	2.27	94.97	3.83
Mean HF duration (s)	12.01	1.79	11.62	2.43	12.54	1.83	12.29	2.41
Last HF duration (s)	15.45	3.74	15.20	5.20	16.98	4.03	17.77	5.93
Mean pattern duration (s)	6.15	1.34	5.82	1.70	5.45	0.93	5.37	1.15
Movement frequency	1.82	0.17	1.81	0.20	1.92	0.15	1.89	0.22
Rotation frequency (degree/s)	41.77	10.01	40.63	12.64	44.95	10.07	44.43	17.73
Connection frequency	0.08	0.10	0.06	0.12	0.14	0.14	0.10	0.15
Leg height index (%)	30.77	3.12	31.17	3.98	32.75	2.23	32.55	2.00
Leg angle deviation (degree)	5.34	2.33	4.94	2.94	4.32	1.75	4.02	1.99

*SD* Standard deviation, *IQR* Interquartile range.

### Multiple liner regression analyses

The results of multiple liner regression analysis revealed a significant regression equation [F (8,96) = 42.694, *p *< 0.001, R^2^ = 0.762]. Mean pattern duration (*p* = 0.001), movement frequency (*p* < 0.001), rotation frequency (*p* = 0.003), leg height index (*p* < 0.001) and leg angle deviation (*p* < 0.001) significantly predicted the total score. The remaining variables (mean HF duration, last HF duration, connection frequency) were not statistically significant predictors to the model (all *p* > 0.05, Table 3). The total score increased with higher last HF duration, movement frequency, rotation frequency, connection frequency, leg index and less mean pattern duration and leg angle deviation.

**Table 3:** Regression coefficients and collinearity for key variables of 105 international teams in recent 11 international official competitions.

Variable	*r*	β	*p*	95% confidence interval for beta		Tolerance	VIF
				Lower	Upper		
Mean HF duration	0.099	− 0.013	0.833	− 0.542	0.438	0.571	1.750
Last HF duration	0.224	0.022	0.741	− 0.202	0.283	0.533	1.876
Mean pattern duration	− 0.470	− 0.190	0.001*	− 1.551	− 0.425	0.766	1.305
Movement frequency	0.604	0.345	< 0.001*	9.731	18.867	0.742	1.347
Rotation frequency	0.249	0.149	0.003*	0.036	0.171	0.964	1.038
Connection frequency	0.358	0.077	0.154	− 2.079	13.002	0.792	1.263
Leg height index	0.743	0.393	< 0.001*	0.595	1.166	0.553	1.810
Leg angle deviation	− 0.537	− 0.229	< 0.001*	− 0.997	− 0.372	0.823	1.216

*r* Correlation coefficient, *β* Standardized regression coefficient, *VIF* Variance inflation factor.

*p < 0.05.

### Comparison of Beijing team and different levels’ international teams

**Figure 4:**
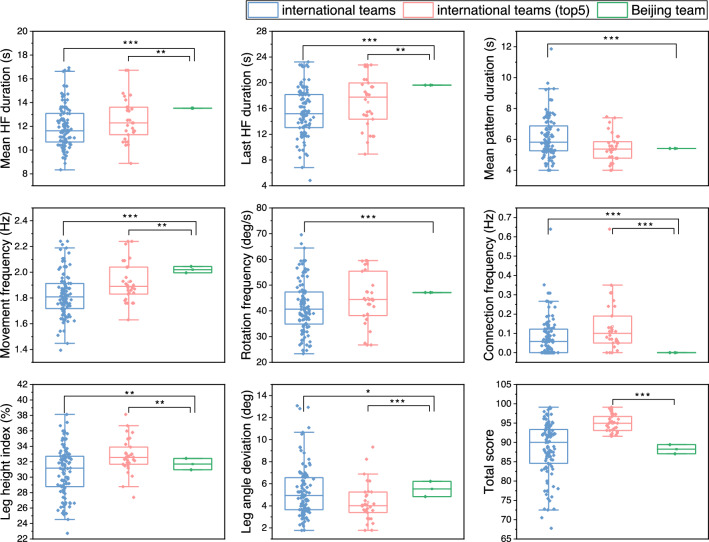
Total scores and 8 key variables of IT, IT-top 5 and BT. Significant differences between groups: ^*^P < 0.05, ^**^P < 0.01, ^***^P < 0.001.

Figure 4 shows the total scores and key variables of international teams (referred to as IT), international teams (top 5) (referred to as IT-top 5) and the trials of Beijing team (referred to as BT). There was no significant difference between BT and IT on total score (Z = 8.895, *p *= 0.313), whereas BT was significantly less than IT-top 5 (Z = 4.782, *p *< 0.001). BT had 6 variables significantly greater than IT, which are mean HF duration (Z =  − 6.656, *p *< 0.001), last HF duration (Z =  − 7.891, *p *< 0.001), movement frequency (Z =  − 7.843, *p *< 0.001), rotation frequency (Z = 8.895, *p *< 0.001), leg angle deviation (Z =  − 2.099, *p *= 0.036) and leg height index (Z =  − 2.638, *p *= 0.008). BT was significantly less than IT on mean pattern duration (Z = 3.953, *p *< 0.001) and connection frequency (Z = 7.323, *p *< 0.001). BT had 4 variables significantly greater than IT-top 5, which are mean HF duration (Z =  − 2.644, *p *= 0.008), last HF duration (Z =  − 3.014, *p *= 0.003), movement frequency (Z =  − 7.843, *p *= 0.004) and leg angle deviation (Z =  − 3.240, *p *< 0.001). Moreover, BT was significantly less than IT-top 5 on connection frequency (Z = 4.544, *p *< 0.001), leg height index (Z = 2.643, *p *= 0.008). There was no significant difference between BT and IT-top 5 on mean pattern duration (Z =  − 0.730, *p *= 0.943) and rotation frequency (Z = 4.783, *p *= 0.299).

## Discussion

This study had 2 parts. Firstly, it aimed to ascertain the significance of hybrid figure (HF) elements in predicting total scores in international artistic swimming competitions. The results showed that 5 HF variables could predict the total score, including leg height index, movement frequency, rotation frequency, leg angle deviation and mean pattern duration, while the remaining variable, including HF duration, last HF duration, connection frequency were not significant predictors. It should be noted that the model still exhibits approximately 30% unexplained variance. Our study primarily focused on investigating the hybrid figures in the team free routine, excluding acrobatics and upper body movements. Furthermore, while the distance covered during leg combinations is also an important factor influencing the overall score, it was not included in the scope of this study due to the inability to accurately measure this parameter from the videos. Second, this study probed the disparities in the HF variables between teams at varying competency levels. The result showed no significant difference between IT and BT on total score, while they were both significantly inferior to IT-top 5. BT surpassed IT-top 5 in terms of mean HF duration, last HF duration, and movement frequency, but lagged behind in connection frequency, leg height index and leg angle deviation. Based on the HF elements from the team free routine trials of BT, the mean HF duration, the last HF duration and the movement frequency are excessive. However, there is a significant deviation in leg angles, and a discernible lack of connection movements, with potential for improvement in leg height.

### HF variables in international teams

In the international competitions analyzed in this study, 8 HF variables were chosen for analysis. According to the FINA rules^5^, mean HF duration, last HF duration, movement frequency and pattern frequency are important variables for difficulty score, while artistic impression score is greatly impressed by rotation frequency and connection frequency. These 6 variables were selected by coaches and swimmers and were confirmed, which could be categorized as HF choreography. The execution of HF is determined by swimmer’s on-the-spot performance, which can be reflected by leg height index and leg angle deviation. Enhancements in leg index, movement frequency, rotation frequency, connection frequency, and last HF duration, couple with reductions in mean pattern duration and leg angle deviation, may correlate with improvements of the total score.

The results showed that five HF variables (Table 2) were significant predictors for total score. The leg height index quantified the elevation of the thigh above water, representing the swimmer's ability to support the body on a high position out of the water, because leg height cannot be directly measured. Greater elevation can provide more striking visual impact, influencing judges’ and spectators’ perceptions^2^. Our findings indicate that in international artistic swimming competitions, the average height of the swimmer's thigh constitutes approximately one-third of the total elevation above the water’s surface, and in fact, top-ranked teams have performed well as reflected by this variable. Performing an excellent elevation during figures requires substantial strength, power, and muscular endurance, which means high exercise intensity^16^. Leg angle deviation also significantly predicts the total score. Swimmers should have enough strength and stability to accomplish precise degree of movements^16^. This element also reflects the synchronization levels of swimmers. In addition, movement frequency is an important HF variable. Higher movement frequency means one should accomplish more elements in a limited period. A previous study^18^ argued that a high movement frequency is related to enhanced performance in international events. Higher movement frequency in artistic swimming competition leads to increased metabolic demand, crucial for monitoring training loads and strategizing performance enhancement^19^. Moreover, both pattern duration and rotation frequency serve as critical HF elements in team free routines. Approximately 50 patterns are typically incorporated into a choreographed performance, with some leading teams executing pattern alterations approximately every 5 seconds^20^. When devising HF elements, coaches and athletes should meticulously evaluate and integrate an adequate number of rotational movements and patterns to optimize performance.

There are three variables could not predict total score, including mean HF duration, last HF duration, and connection frequency. During international competitions, the team free routines usually last 3–4 min and artistic swimmers may spend 50–65% of the total time with their faces underwater^10,11^. A study investigated the relationship between sport-specific test and artistic swimming total score, showing that the ability to swim underwater continuously was significantly correlated to the competition score^21^. However, the current study showed that the duration of HF was not a significant predictor for the total score in the international competitions, which may indicate that the length of HF time is not the most crucial aspect of the HF performance. A previous study showed that increased difficulty of figures would decrease the duration of underwater, because the energy expenditure will be greater^11^. Rapid tachypneic and dyspneic neural stimuli are directly proportional to the duration underwater and the intensity of the exercise involved^22^. Taking into account that artistic swimming competitions involve numerous prolonged periods of apnea underwater, such events impose substantial demands on both the aerobic and anaerobic energy systems of swimmers^23–25^. Increased difficulty would decrease the duration of underwater, because the energy expenditure required for difficult figures is greater^11^. The current study underscores that, to secure desirable scores in artistic swimming team free routines, swimmers should attain high movement frequencies within constrained timeframes, which emphasizes the anaerobic metabolic demands placed on swimmers. While last HF duration was not a significant predictor for the total score, we found most teams likely to schedule prolonged HF towards the competition’s conclusion.

### Comparison of different levels’ teams

In the second part of this study, we compared the Beijing team’s total score and HF variables with those of teams in international official competitions. In the results of total scores, there was no significant difference between BT and IT, but BT’s total score was significantly lower than that of IT-top 5. For the key HF variables, the result showed that the leg height index of BT was significantly lower than IT-top 5, while the movement frequency and leg angle deviation were significantly higher than those in IT-top 5. These findings suggest that Beijing artistic swimming team may place excessive emphasis on the choreography difficulty, while their execution of movements should be improved. Previous research indicated that the difficulty and combination of high-intensity exercise may affect the activation of glycogenolysis in the muscles^26^. Recent study in artistic swimming has demonstrated that blood lactate accumulation is significantly influenced by extensive muscle movements accompanied by periods of apnea during competitions^27^, which indicates a great anaerobic energy requirement. Metabolic reactions can lead to a significant acidic environment within the body, which may affect the performance during competitions^28^. In artistic swimming competitions, more frequent high-intensity exercise may accelerate the consumption of peripheral O_2_, leading to tissue hypoxia and increased lactate production^23,29^. These changes negatively affect the different muscle cell organelles that are involved in the transmission of neuronal signals and produce fatigue^30^, which could cause a decline of performance^28,31^. In this regard, we speculate that excessive movement frequency may not always facilitate the improvement of total score. Such a scenario might significantly escalate movement difficulty, ultimately compromising the quality of execution.

According to the results of the first part of this study for the international official competitions, mean HF duration, last HF duration and connection frequency were not significant predictors of total score. It is worth noting that BT had significantly higher mean HF duration and last HF duration than IT and IT-top 5. However, the total score of BT was significantly lower than that of IT-top 5. In artistic swimming, some coaches and swimmers hold the training philosophy of "the more, the better" and may arrange excessive training load for movements^32^. In team sports, reasonable arrangement and adjustment of exercise intensity ensure that sufficient energy could be reserved to respond to competition demands^33,34^. Overly long HF duration combined with an excessive movement frequency of Beijing team may lead to negative effects on performance. Previous study suggested that coaches should carefully arrange the length of HF and the intensity of the movement when designing a routine^35^. In addition, the connection frequency of BT is significantly lower than that of both IT and IT-top 5. This factor may have potentially impact on the total score. The latest artistic swimming rules^36^ underscore the importance of connection movements. The modified total score calculation method in the new rules^37^ entails multiplying the degree of difficulty and execution level, and then adding the artistic performance score to obtain the total score. This places greater emphasis on balancing HF choreography difficulty and movement execution quality, acknowledging that these are interconnected aspects of performance.

When designing hybrid figures choreography of team free routine in artistic swimming, coaches should prioritize appropriate movement frequency, pattern changes and rotations rather than focusing on excessively long durations. Moreover, swimmers should strive for the highest possible leg height and precise angle to ensure superior movement execution quality. While excellent choreography plays a crucial role in achieving a high total score, prioritizing movement execution quality remains paramount. Consequently, the findings from this study may provide useful guidelines for artistic swimming coaches when devising HF choreography and tailored training plans.

### Limitations

Due to the varied compositions of teams and events, there are differences in the number and position of movements of vertical position with one or two legs. As this paper present, the index of the completion quality of leg movements was only analyzed for the first movement, not for all movements. In future studies, more data can be included for analysis. In addition to the crucial HF elements that were analyzed in this research, the overall distance traversed by the swimmers in the water and the formation area (the size of the gap between team members) could also have a certain degree of impact to the total score. However, due to limited video and equipment resources, these data could not be calculated and included in current study. Another limitation is the judges’ level for the Beijing team, which may not be same as that in the international official competitions. Future studies are recommended to use a group of international-level judges.

## Conclusions

In international artistic swimming team free routines, HF elements, including leg height index, movement frequency, rotation frequency, leg angle deviation, and mean pattern duration were significant predictors for the total scores. HF duration was not a significant predictor and has been removed as a primary judging criterion for artistic swimming. Beijing team’s total score was significantly lower than the international teams (top 5). For leg height index and leg angle deviation, international teams (top 5) displayed a significantly better performance than Beijing team. While Beijing team was significantly higher than those in international teams (top 5) on movement frequency, mean HF duration and last HF duration. Both the difficulty of the choreography and the quality of movement execution should be taken into consideration when designing HF choreography for artistic swimming team free routines, as excessive prolonged underwater breath-holding and intensity of movements may lead to a decline in athletic performance of swimmers during the competition.

## Data Availability

The datasets generated during and analyzed during the current study are available from LY on reasonable request.
