# Author Correction: Maximizing choreography and performance in artistic swimming team free routines: the role of hybrid figures

**DOI:** 10.1038/s41598-024-66241-4

**Published:** 2024-07-08

**Authors:** Leijiao Yue, Jiawen Zhang, Wenlai Cui, Rui Yang, Jun Yin

**Affiliations:** 1 Physical Education and Training Institute, Capital University of Physical Education and Sports, Beijing, China; 2 School of Swimming and Diving, Beijing Sport University, Beijing, China; 3 School of Dance and Martial Arts, Capital University of Physical Education and Sports, Beijing, China

Correction to: *Scientific Reports* 10.1038/s41598-023-48622-3, published online 02 December 2023

The Funding section in the original version of this Article was omitted. The Funding section now reads:

“This work was supported by the Emerging Interdisciplinary Platform for Medicine and Engineering in Sports (EIPMES).”

The original Article has been corrected.

